# Trends in Selected Chronic Conditions and Behavioral Risk Factors Among Women of Reproductive Age, Behavioral Risk Factor Surveillance System, 2001-2009

**Published:** 2011-10-15

**Authors:** Donald K. Hayes, Amy Z. Fan, Ruben A. Smith, Jennifer M. Bombard

**Affiliations:** Hawaii Department of Health, Family Health Services Division. Dr Hayes is also affiliated with the Centers for Disease Control and Prevention, Atlanta, Georgia; Centers for Disease Control and Prevention, Atlanta, Georgia; Centers for Disease Control and Prevention, Atlanta, Georgia; Centers for Disease Control and Prevention, Atlanta, Georgia

## Abstract

**Introduction:**

Some potentially modifiable risk factors and chronic conditions cause significant disease and death during pregnancy and promote the development of chronic disease. This study describes recent trends of modifiable risk factors and controllable chronic conditions among reproductive-aged women.

**Methods:**

Data from the 2001 to 2009 Behavioral Risk Factor Surveillance System, a representative state-based telephone survey of health behavior in US adults, was analyzed for 327,917 women of reproductive age, 18 to 44 years. We calculated prevalence ratios over time to assess trends for 4 selected risk factors and 4 chronic conditions, accounting for age, race/ethnicity, education, health care coverage, and individual states.

**Results:**

From 2001 to 2009, estimates of 2 risk factors improved: smoking declined from 25.9% to 18.8%, and physical inactivity declined from 25.0% to 23.0%. One risk factor, heavy drinking, did not change. From 2003 to 2009, the estimates for 1 risk factor and 4 chronic conditions worsened: obesity increased from 18.3% to 24.7%, diabetes increased from 2.1% to 2.9%, high cholesterol increased from 10.3% to 13.6%, asthma increased from 13.5% to 16.2%, and high blood pressure increased from 9.0% to 10.1%. All trends were significant after adjustment, except that for heavy drinking.

**Conclusion:**

Among women of reproductive age, prevalence of smoking and physical inactivity improved, but prevalence of obesity and all 4 chronic conditions worsened. Understanding reasons for the improvements in smoking and physical activity may support the development of targeted interventions to reverse the trends and help prevent chronic disease and adverse reproductive outcomes among women in this age group.

## Introduction

In the United States, 53.5% of deaths in women of reproductive age were due to unintentional injuries, cancer, and heart disease in 2007 (1). In 2008, women of reproductive age incurred health care expenses estimated at $170.4 billion, or 14.8% of all health care expenditures, in the United States (2). Cancer and heart disease are the second and third leading causes of death in this population, but little attention has focused on these women's needs for primary prevention of chronic disease outside of routine screening for cancer (3,4). Many of the same potentially modifiable risk factors for cancer are also risk factors for heart disease and stroke, such as smoking, obesity, physical inactivity, and heavy drinking (5,6). Hypertension, diabetes, asthma, and high cholesterol are also associated with these risk factors (7,8). Additionally, these risk factors and chronic conditions are related to adverse reproductive health outcomes, including cesarean delivery, eclampsia, perinatal infections and complications, preterm delivery, low birth weight, and infant death (9-13). Preventing and managing these risk factors and conditions in women of reproductive age may improve pregnancy outcomes and the overall health of women.

Recent studies show increases in risk factors for heart disease, diabetes, and cancer in the general population (14-16), but only identified 1 study for women of reproductive age (17). Ahluwalia et al highlighted worsening estimates for obesity, smoking, high blood pressure, and diabetes in prevalence estimates from 1991-1992 to 2000-2001 among women of reproductive age (17). The surveillance of trends among women of reproductive age in indicators related to adverse birth outcomes and development of chronic disease can identify groups at increased risk and guide public health prevention and management efforts. The objective of this study was to describe recent trends of modifiable risk factors and controllable chronic conditions among women of reproductive age.

## Methods

We performed a cross-sectional analysis using data from the Behavioral Risk Factor Surveillance System (BRFSS), a state-based, random-digit–dialed telephone survey of the US noninstitutionalized, civilian population aged 18 years or older. In particular, we analyzed self-reported data from 341,989 women of reproductive age (18-44 y), who participated in the 2001, 2003, 2005, 2007, and 2009 BRFSS surveys and lived in 1 of the 50 states or the District of Columbia. Data from odd years were selected for this analysis because questions related to risk factors and chronic conditions are asked only in odd years. The Council of American Survey Research Organizations (CASRO) response rate reflects both telephone sampling efficiency and the degree of participation among eligible respondents contacted (18). In 2009, the median CASRO rate was 53% and ranged from 38% to 67%. The median CASRO rates were 51%, 53%, 51%, and 51% in 2001, 2003, 2005, and 2007, respectively, with similar ranges. The cooperation rate reflects the proportion who completed an interview among eligible people contacted. The median cooperation rate for BRFSS in 2009 was 75% and ranged from 55% to 88%. The median cooperation rates were 71%, 75%, 75%, and 72% in 2001, 2003, 2005, and 2007, respectively. Relative to other surveys, data from BRFSS have acceptable reliability and validity for several chronic disease risk factors and conditions (19,20). Detailed information on the sampling methodology, survey weighting procedures, quality assurance of the survey, and other aspects of this survey is available online (www.cdc.gov/brfss/index.htm).

### Outcomes

We examined 4 risk factors (smoking, physical inactivity, heavy drinking, and obesity) and 4 chronic conditions (chronic high blood pressure, high cholesterol, chronic diabetes, and asthma) among women of reproductive age. Smoking was defined by self-report of smoking at least 100 cigarettes in one's lifetime and still smoking at the time of the survey. Current physical inactivity was defined as a response of no to the question: "During the past month, other than your regular job, did you participate in any physical activities or exercises such as running, calisthenics, golf, gardening, or walking for exercise?" Heavy drinking was defined by self-reported daily alcohol consumption of more than 1 drink per day in the past 30 days. Obesity was defined as having a body mass index (BMI) of at least 30.0 kg/m^2^ based on self-reported weight and height. The chronic conditions (chronic high blood pressure, high cholesterol, chronic diabetes, and asthma) were considered present if the woman reported she had ever been told by a doctor or other health professional that she had the condition. For high blood pressure, we restricted the analysis from 2003 to 2009 because a substantially different question related to high blood pressure was asked in the 2001 survey. For all other measures, 2001 to 2009 was the period assessed.

### Covariates

We categorized age into 3 groups (18-24, 25-34, and 35-44 y). Self-identified race/ethnicity included non-Hispanic white, non-Hispanic black, Hispanic (any race), Asian, American Indian or Alaska Native (AI/AN), or all others. The "all others" group represented just over 3% of the weighted population of women of reproductive age for each year, and analysis excluding them did not significantly change the conclusions. Therefore, they were maintained as their own group to increase sample size of the study. Education levels were based on highest grade or year of school completed and were categorized as not completing high school (<12 y), completing high school or its equivalent (12 y), some college course work, or college graduate or more. Access to health care was defined as having any health care coverage on the basis of the question "Do you have any kind of health care coverage, including health insurance, prepaid plans such as HMOs, or government plans such as Medicare?" Because of possible variation in risk factors or chronic conditions related to state of residence, a variable representing each state was included as a covariate in the analysis.

### Exclusions

A total of 341,989 women of reproductive age were surveyed over these 5 periods. Exclusion criteria included women pregnant at the time of the survey (4.1%, n = 14,072). Pregnant women are likely to change their behaviors and report different risks, particularly smoking and alcohol intake during pregnancy, so we chose to exclude them from the analyses. This resulted in an overall sample size of 327,917. Women with missing information on any risk factor, chronic condition, or covariates were excluded from specific analyses. Missing information ranged from 0.05% for diabetes (n = 179) to 6.4% for obesity (n = 21,148).

### Statistical analysis

Annual prevalence estimates and 95% confidence intervals (CIs) were calculated for each risk factor and chronic condition. Estimates were plotted over the 5 periods, except for high blood pressure, which was only measured for 4 periods. An approach using predicted marginals estimated prevalence ratios for each risk factor and chronic condition; survey year was the primary variable, following recommendations for complex national surveys (21). We evaluated the overall trend for significance, accounting for differences in the individual covariates of age, race/ethnicity, education, health care coverage, and a variable representing each state. Data were weighted to reflect each state's non-institutionalized civilian population. SAS version 9.2 and SAS-callable SUDAAN version 10.0 (SAS Institute, Inc, Cary, North Carolina) were used to account for the complex sampling design in order to provide population estimates and calculate accurate variance estimates.

## Results

### Selected population estimates

The distribution for all sociodemographic population estimates remained unchanged over time, with a few exceptions: lower estimates in women aged 18 to 24 years in 2007 and 2009 compared to earlier years and a consequent increase in those aged 25 to 34 in 2007 and 2009 and aged 35 to 44 in 2007; respondents included a larger proportion of Hispanics in 2005, 2007, and 2009 compared to earlier years; and a larger proportion of college graduates in 2007 and 2009 compared to earlier years (Table 1).

### Changes in risk factors

Among risk factors studied, unadjusted estimates of obesity worsened over time, while improvements were observed for smoking, physical inactivity, and heavy drinking (Table 2). The unadjusted prevalence estimate of obesity among women of reproductive age worsened from 18.3% in 2001 to 24.7% in 2009. From 2001 to 2009, the unadjusted prevalence estimate of smoking improved from 25.9% to 18.8%, current physical inactivity from 25.0% to 23.0%, and heavy drinking from 5.5% in 2001 to 4.9%. After adjusting for age, race/ethnicity, education, health care coverage, and individual state, the estimate in 2009 was significantly different from 2001 for all risk factors except for heavy drinking (Table 3).

### Changes in chronic conditions

Among the chronic conditions studied, unadjusted estimates of asthma, high cholesterol, chronic diabetes, and chronic high blood pressure worsened over time. The unadjusted prevalence estimate of asthma among women of reproductive age worsened from 13.5% in 2001 to 16.2% in 2009 (Table 2). From 2001 to 2009, the unadjusted prevalence of high cholesterol worsened from 10.3% to 13.6%, and chronic diabetes worsened from 2.1% to 2.9%. From 2003 to 2009, chronic high blood pressure worsened from 9.0% to 10.1%. After adjusting for age, race/ethnicity, education, health care coverage, and individual state, the estimate in 2009 was significantly different from the baseline estimate for all chronic conditions (Table 3).

## Discussion

This study on the trends of common risk factors and chronic conditions among women of reproductive age revealed both positive and concerning trends since 2001. In particular, the benefits related to declines in smoking and current physical inactivity have occurred in the face of worsening trends for obesity, chronic high blood pressure, chronic diabetes, high cholesterol, and asthma. This is of particular concern for women of reproductive age and their children, who are at increased risk because of the effect on reproductive health outcomes and long-term development of chronic disease and its complications over time. The promotion of healthy lifestyle choices that can prevent or delay the development of disease among women of reproductive age may improve reproductive health outcomes, decrease the development of and burden of chronic disease, and improve health throughout the life course.

Our study extends the work of Ahluwalia et al another 10 years by highlighting the estimates related to several risk factors and chronic disease (17). Additionally, we used multivariate analyses to control for 5 covariates that potentially influence the observed trends. Overall, our study showed worsening measures of obesity, high blood pressure, diabetes, high cholesterol, and asthma from 2001 to 2009 among women of reproductive age but also highlights improvement in smoking and current physical activity. These trends among reproductive-aged women are similar to those seen in the general US population (14-16). In the general population, declines in smoking are generally attributed to increased taxation, increased awareness of the dangerous health effects of smoking, and policies that limit smoking in public places (22). These approaches are likely also influencing the declines in smoking among women of reproductive age, and continued emphasis on the dangers related to both reproductive health and overall chronic disease prevention are needed. The lack of a change in heavy drinking is concerning, particularly with the increased awareness of adverse reproductive health attributed to alcohol. Further clarifying the reasons why women of reproductive age continue to consume more than 1 drink daily may identify potential programs to increase awareness of its effect on both reproductive health and chronic disease prevention. The worsening estimates of obesity are alarming for women of reproductive age and the general US population. Increased understanding and population-level approaches are needed to combat this growing public health problem. The worsening trends in chronic conditions in the general population are likely related to increased rates of poor nutrition and decreased activity-related lifestyles (23). Our study found a somewhat different picture for chronic conditions, with decreased levels of physical inactivity over time but worsening measures of all chronic conditions. Worsening obesity may be substantially contributing to the increases in the chronic conditions. Additional analysis to evaluate the effect of rising obesity may further clarify its contribution to worsening of overall chronic disease trends. Surveillance data will be necessary to monitor the effect of worsening chronic disease trends on both reproductive health and chronic disease prevention.

The findings in this report are subject to at least 3 limitations. First, BRFSS is a telephone-based survey that documents self-reported information and is subject to bias from recall and the likelihood for a socially desirable response (eg, underreporting actual weight, not currently smoking, overstating activity) (24). Second, current physical inactivity could be under- or overestimated because this study only looked at leisure-time physical activity, rather than a more specific measure, such as the proportion of those meeting recommended daily requirements for moderate or vigorous activity as encouraged by the Surgeon General (25). More specific measures for the other variables were not available in the data, so we chose to limit the physical activity questions to the general leisure-time physical inactivity measure. A third limitation is that neither the relative effect of the particular risk factors or chronic conditions on overall health nor the severity of the risk factors or the level of treatment for the chronic conditions were included even though some may have a greater effect on primary prevention of chronic disease or reproductive outcomes than others.

The purpose of this article was to use population-based survey data to highlight trends among women of reproductive age for several common risk factors and chronic conditions that affect reproductive health and chronic disease and its complications. Surveillance of risk factors and chronic conditions related to chronic disease can ensure that population-based approaches are reaching particular subgroups such as women of reproductive age, or highlight that more targeted approaches may be needed. Variation in these trends among different populations such as age or race/ethnicity should also be assessed. Evaluating and monitoring trends in adverse birth outcomes and the development of chronic disease associated with these risk factors and chronic conditions are needed. Individual, community, and population approaches among women of reproductive age should consider both the immediate consequences related to reproductive outcomes and the lifetime risks associated with chronic disease.

## Figures and Tables

**Table 1:** Characteristics of Adult Women of Reproductive Age, BRFSS, 2001-2009

Characteristic	Year, % (95% CI)^a^				

	2001 (n = 53,965)	2003 (n = 60,974)	2005 (n = 73,863)	2007 (n = 75,346)	2009 (n = 63,769)
**Age, y**					
18-24	23.6 (22.9-24.3)	24.1 (23.4-24.8)	24.8 (24.1-25.5)	20.6 (19.9-21.3)	22.2 (21.5-23.0)
25-34	34.2 (33.5-34.8)	34.0 (33.4-34.7)	34.5 (33.9-35.2)	36.5 (35.8-37.2)	37.4 (36.7-38.1)
35-44	42.3 (41.6-43.0)	41.9 (41.2-42.6)	40.7 (40.1-41.3)	43.0 (42.3-43.6)	40.4 (39.8-41.1)
**Race/ethnicity**					
Non-Hispanic white	66.5 (65.8-67.2)	64.3 (63.6-65.1)	63.6 (62.9-64.3)	63.0 (62.3-63.8)	62.3 (61.6-63.1)
Non-Hispanic black	11.5 (11.1-11.9)	12.1 (11.6-12.6)	11.6 (11.2-12.0)	11.4 (11.0-11.8)	11.8 (11.3-12.3)
Hispanic (any race)	14.9 (14.2-15.5)	16.0 (15.4-16.7)	17.6 (17.0-18.3)	17.9 (17.2-18.5)	18.1 (17.5-18.8)
Asian	3.0 (2.6-3.3)	3.1 (2.8-3.5)	3.0 (2.7-3.3)	3.4 (3.1-3.7)	3.7 (3.4-4.0)
American Indian/Alaska Native	1.1 (0.9-1.2)	1.1 (1.0-1.3)	1.1 (1.0-1.2)	1.2 (1.0-1.4)	1.0 (0.9-1.1)
All others	3.2 (3.0-3.5)	3.3 (3.0-3.6)	3.1 (2.9-3.4)	3.3 (3.0-3.6)	3.1 (2.9-3.4)
**Education**					
<High school	10.5 (9.9-11.0)	10.6 (10.1-11.1)	11.3 (10.8-11.8)	10.4 (9.9-10.9)	10.4 (9.9-10.9)
High school or equivalent	28.8 (28.2-29.4)	27.8 (27.1-28.5)	27.3 (26.7-28.0)	25.9 (25.2-26.5)	24.3 (23.6-24.9)
Some college	31.3 (30.7-31.9)	30.4 (29.7-31.0)	29.3 (28.7-29.9)	28.9 (28.3-29.6)	29.4 (28.7-30.1)
College graduate	29.5 (28.9-30.2)	31.3 (30.7-31.9)	32.0 (31.4-32.6)	34.8 (34.2-35.5)	35.9 (35.3-36.6)
**Health care coverage**					
Yes	81.8 (81.2-82.4)	80.2 (79.6-80.8)	79.6 (79.0-80.2)	80.0 (79.4-80.6)	79.9 (79.3-80.5)
No	18.2 (17.6-18.8)	19.8 (19.2-20.4)	20.4 (19.8-21.0)	20.0 (19.4-20.6)	20.1 (19.5-20.7)

Abbreviations: BRFSS, Behavioral Risk Factor Surveillance System; CI, confidence interval.

a Estimates and CIs are weighted.

**Table 2:** Prevalence of Risk Factors and Chronic Conditions, by Year, Among Adult Women of Reproductive Age, BRFSS, 2001-2009

Risk Factor/Chronic Condition	Year, % (95% CI)^a^				

	2001	2003	2005	2007	2009
**Risk factor^b^**					
Obesity	18.3 (17.7-18.9)	19.5 (18.9-20.1)	21.6 (21.0-22.2)	23.4 (22.8-24.0)	24.7 (24.1-25.3)
Physical inactivity	25.0 (24.3-25.6)	22.6 (21.9-23.2)	23.5 (22.9-24.1)	22.4 (21.8-23.1)	23.0 (22.4-23.6)
Smoking	25.9 (25.3-26.5)	24.1 (23.5-24.7)	22.3 (21.7-22.8)	20.4 (19.9-21.0)	18.8 (18.3-19.4)
Heavy drinking	5.5 (5.1-5.9)	5.8 (5.5-6.2)	4.9 (4.6-5.2)	4.9 (4.6-5.2)	4.9 (4.6-5.3)
**Chronic condition^c^**					
Asthma	13.5 (13.0-14.0)	14.1 (13.6-14.6)	15.1 (14.6-15.6)	15.2 (14.8-15.8)	16.2 (15.6-16.7)
High cholesterol	10.3 (9.9-10.7)	11.8 (11.4-12.3)	12.1 (11.7-12.6)	12.7 (12.3-13.2)	13.6 (13.2-14.1)
Chronic diabetes	2.1 (1.9-2.3)	2.9 (2.6-3.2)	2.5 (2.3-2.7)	2.9 (2.7-3.1)	2.9 (2.7-3.2)
Chronic high blood pressure	NA^d^	9.0 (8.6-9.4)	9.2 (8.8-9.6)	9.8 (9.4-10.2)	10.1 (9.7-10.5)

Abbreviations: BRFSS, Behavioral Risk Factor Surveillance System; CI, confidence interval; NA, not applicable.

a Estimates and CIs are weighted.

b Obesity defined as body mass index ≥30.0 kg/m^2^. Physical inactivity defined as no physical activity in the previous 30 days. Smoking defined as having smoked ≥100 cigarettes in lifetime and currently smoking. Heavy drinking defined as >1 drink/d.

c Women were categorized as having a chronic condition if they had ever been told by a doctor or other health professional that they had the condition. "Chronic diabetes" excludes gestational diabetes. "Chronic high blood pressure" excludes pregnancy-related hypertension.

d The BRFSS question for high blood pressure in 2001 was substantially different than for the other years, and this value was not included in the analysis.

**Table 3:** Odds Ratios for Risk Factors and Chronic Conditions Among Adult Women of Reproductive Age, BRFSS, 2001-2009

Risk Factor/Chronic Condition	Prevalence Ratios of 2009 Compared to Reference Year^a^		

	Crude OR (95% CI)^b^	AOR (95% CI)^c^	AOR *P* Value^d^
**Risk factor^e^**			
Obesity	1.35 (1.29-1.40)	1.39 (1.33-1.44)	<.001
Physical inactivity	0.92 (0.89-0.95)	0.93 (0.90-0.97)	<.001
Smoking	0.73 (0.70-0.75)	0.79 (0.76-0.82)	<.001
Heavy drinking	0.91 (0.83-1.00)	0.93 (0.85-1.02)	.12
**Chronic condition^f^**			
Asthma	1.20 (1.14-1.26)	1.23 (1.17-1.29)	<.001
High cholesterol	1.33 (1.16-1.40)	1.35 (1.28-1.42)	<.001
Chronic diabetes	1.40 (1.24-1.59)	1.45 (1.28-1.64)	<.001
Chronic high blood pressure	1.12 (1.06-1.19)	1.16 (1.09-1.23)	<.001

Abbreviations: BRFSS, Behavioral Risk Factor Surveillance System; OR, odds ratio; CI, confidence interval; AOR, adjusted odds ratio.

a Individual models were used to assess difference between 2009 and comparison year 2001 except for chronic high blood pressure, which used 2003.

b Confidence interval around the weighted prevalence ratio estimate.

c Individual models for each risk factor and chronic condition were adjusted for age, race/ethnicity, education, health care coverage, and individual state.

d Calculated by using *t* tests for the difference in adjusted marginal predictors used to estimate prevalence ratios.

e Obesity defined as body mass index ≥30.0 kg/m^2^. Physical inactivity defined as no physical activity in the previous 30 days. Smoking defined as having smoked ≥100 cigarettes in lifetime and currently smoking. Heavy drinking defined as >1 drink/d.

f Women were categorized as having a chronic condition if they had ever been told by a doctor or other health professional that they had the condition. "Chronic diabetes" excludes gestational diabetes. "Chronic high blood pressure" excludes pregnancy-related hypertension.
