# Erratum, Vol. 8: Issue 6

**Published:** 2011-12-15

In the article "Trends in Selected Chronic Conditions and Behavioral Risk Factors Among Women of Reproductive Age, Behavioral Risk Factor Surveillance System, 2001-2009," the title and some column heads for Table 3 were incorrect. The title should have read "Prevalence Ratios for Risk Factors and Chronic Conditions Among Adult Women of Reproductive Age, BRFSS, 2001-2009," and the column heads should have read "Crude Ratio," "Adjusted Ratio," and "Adjusted Ratio P Value." The correction was made to our website on October 24, 2011 and appears online at http://www.cdc.gov/pcd/issues/2011/nov/10_0083.htm. We regret any confusion or inconvenience this error may have caused.

